# p53 at the Crossroads between Doxorubicin-Induced Cardiotoxicity and Resistance: A Nutritional Balancing Act

**DOI:** 10.3390/nu15102259

**Published:** 2023-05-10

**Authors:** Yuanfang Guo, Yufeng Tang, Guangping Lu, Junlian Gu

**Affiliations:** 1 School of Nursing and Rehabilitation, Cheeloo College of Medicine, Shandong University, Jinan 250012, China; 201915951@mail.sdu.edu.cn (Y.G.); 202020820@mail.sdu.edu.cn (G.L.); 2 Department of Orthopedic Surgery, The First Affiliated Hospital of Shandong First Medical University, Jinan 250014, China; ttang1987@163.com

**Keywords:** p53, DOX-induced cardiotoxicity, DOX resistance, dietary nutrients, natural products

## Abstract

Doxorubicin (DOX) is a highly effective chemotherapeutic drug, but its long-term use can cause cardiotoxicity and drug resistance. Accumulating evidence demonstrates that p53 is directly involved in DOX toxicity and resistance. One of the primary causes for DOX resistance is the mutation or inactivation of p53. Moreover, because the non-specific activation of p53 caused by DOX can kill non-cancerous cells, p53 is a popular target for reducing toxicity. However, the reduction in DOX-induced cardiotoxicity (DIC) via p53 suppression is often at odds with the antitumor advantages of p53 reactivation. Therefore, in order to increase the effectiveness of DOX, there is an urgent need to explore p53-targeted anticancer strategies owing to the complex regulatory network and polymorphisms of the p53 gene. In this review, we summarize the role and potential mechanisms of p53 in DIC and resistance. Furthermore, we focus on the advances and challenges in applying dietary nutrients, natural products, and other pharmacological strategies to overcome DOX-induced chemoresistance and cardiotoxicity. Lastly, we present potential therapeutic strategies to address key issues in order to provide new ideas for increasing the clinical use of DOX and improving its anticancer benefits.

## 1. Introduction

The clinical application of doxorubicin (DOX) can often be a double-edged sword. DOX mainly exerts its antitumor effects by inserting itself into the DNA helix structure, thereby preventing DNA replication and RNA synthesis [1]. DOX can also interfere with DNA repair and synthesis by inhibiting topoisomerase 2 [2,3]. Furthermore, inside cells, DOX is converted into an unstable intermediate called semiquinone. Semiquinone generates reactive oxygen species (ROS), which cause cell membrane and DNA damage, ultimately inducing apoptosis [4].While DOX is considered one of the most effective drugs for lung, breast, ovarian, uterine, and hematological malignancies, its cumulative dose-limiting toxicity and drug resistance limit its success in anticancer therapy [5]. Owing to its non-specific nature, DOX exerts cytotoxic effects against not only tumors but also healthy tissues. Compared with other organs, cardiac tissue has a low antioxidant capacity and limited regenerative potential. Therefore, it is highly sensitive to DOX-induced oxidative damage, and this cardiotoxicity is usually progressive and irreversible [6]. In the last 50 years, several studies have extensively investigated the mechanisms of DOX-induced cardiotoxicity (DIC), focusing on oxidative stress, endoplasmic reticulum stress, inflammation, calcium dyshomeostasis, and the dysregulation of apoptosis and autophagy [5,7]. Apart from its dose-limiting toxicities, the efficacy of DOX is also limited by inherent or acquired multidrug resistance (MDR), which may lead to tumor recurrence and metastasis [8]. The mechanisms of chemoresistance can be roughly divided into primary and acquired drug resistance. Primary chemoresistance occurs due to the natural resistance of some tumors to DOX and is not a result of treatment pressure [9]. Acquired drug resistance can be of two types: cancer stem cell (CSC)-mediated and non-CSC-mediated. CSC-mediated drug resistance can occur due to the proliferation and differentiation of CSCs, leading to tumor recurrence or metastasis. Non-CSC-mediated drug resistance mechanisms include increased DOX efflux due to multidrug resistance proteins (MRPs) 1–9 [10], increased DNA damage repair [11], apoptosis escape [12], and autophagy imbalance [13].

Over the years, the underlying causes of DIC and chemoresistance have been the subjects of extensive research. In particular, the tumor suppressor gene p53 has garnered much attention due to its crucial role in coordinating the intricate regulatory network in the tumor environment. By activating p53 and its target genes (p21, BAX, and PUMA), DOX promotes DNA repair, cell cycle arrest, and tumor cell death [14]. However, p53 activation can also cause non-specific chemotoxicity in normal cells. In addition, the mutation or inactivation of p53 in most human cancers causes DOX resistance and accelerates tumor development [15]. Therefore, restoring the tumor-inhibitory function of wild-type p53 (wtp53) has become an appealing anticancer strategy. Paradoxically, p53 inhibition also appears to be beneficial in a variety of DIC models, although it is obviously not effective in preventing chemotherapeutic resistance.

Several new dietary nutrients, natural products, and drugs that can simultaneously boost the sensitivity of DOX and reduce its adverse effects have attracted attention. In this review, we provide a new perspective on enhancing the efficacy of DOX chemotherapy by summarizing the intricate role of p53 in this process. Further, we highlight the development of anticancer strategies that target p53 to limit DIC and overcome chemoresistance and the challenges associated with these strategies.

## 2. Mechanisms of p53 in DIC

**Figure 1:**
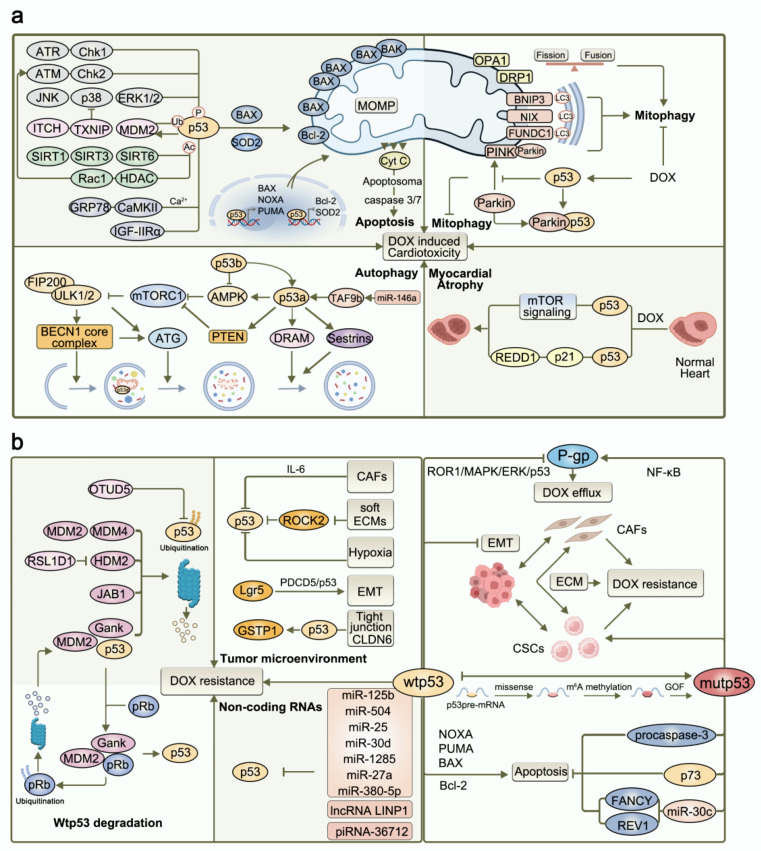
(**a**) Mechanism of action of p53 in DOX-induced cardiotoxicity. p53 is activated by DOX-induced DNA damage and reactive oxygen species accumulation, which leads to the development of cardiotoxicity by inducing cardiomyocyte apoptosis, regulating autophagy, inhibiting mitochondrial autophagy, and causing cardiac atrophy. (**b**) Mechanism of p53 in DOX resistance. p53 is frequently mutated, inhibited, or degradation inactivated in tumor tissues, leading to DOX resistance.

p53 has been identified as a key transcriptomic regulator of DIC in a transcriptomic profiling study [16]. Moreover, p53-induced cell death receptor upregulation has been proposed to play a role in regulating cardiomyocyte apoptosis and autophagy and in inducing myocardial atrophy (Figure 1a).

### 2.1. p53 Induces Cardiomyocyte Apoptosis

Under physiological conditions, p53 is maintained at low levels via proteasomal degradation [17]. The expression of p53 increases during stress injury in the heart through transcription-dependent and -independent pathways. The transcription-dependent pathways involve the regulation of mitochondrial apoptotic signals, including the upregulation of pro-apoptotic genes such as PUMA, NOXA, and BAX and the downregulation of anti-apoptotic genes such as Bcl-2 [18]. The transcription-independent effects involve direct interactions with factors such as BAX [19] and superoxide dismutase 2 (SOD2) [20]. BAX induces cardiomyocyte apoptosis through a caspase cascade mediated by the mitochondrial release of cytochrome C (Cyt C). SOD2 interacts with p53, leading to a decrease in superoxide anion scavenging and mitochondrial membrane potential, which results in the activation of apoptosis.

Several studies have demonstrated that DOX mediates cardiomyocyte apoptosis by activating p53 transcriptional activity in the mitochondria. p53 overexpression can also induce apoptosis through its transactivation in the autologous nucleus [21]. This pro-apoptotic activity has been implicated in the relationship between the dose- and treatment duration-dependent effects of DOX and the status of the p53 gene. Higher DOX concentrations mainly induce p53-dependent cell apoptosis, while lower DOX concentrations cause cardiotoxicity via bioenergetic dysfunction [22]. p53 suppression prevents acute cardiotoxicity following DOX treatment. In contrast, inhibiting p53 activity can increase cardiomyocyte apoptosis during the late stage of DOX treatment. Notably, p53 regulates the normal transcription of genes involved in cardiac mitochondrial biogenesis and bioenergetics, and it plays a complex role in DIC [23,24]. In DOX-induced mouse models, p53 mutants (R172H) lack the typical tumor-suppressor effects of p53, such as apoptosis. However, mitochondrial biogenesis is preserved, and cardiac dysfunction is prevented [25]. Further, p53 has similar protective effects for mitochondrial DNA in cardiomyocytes derived from DOX-treated human induced pluripotent stem cells (iPSCs) [25].

### 2.2. p53 Regulates Cardiac Autophagy and Mitophagy

Autophagy is generally considered a mechanism for the recycling of intracellular components, and excessive autophagy is believed to be maladaptive and detrimental. At present, the mechanism of autophagy induction in the heart is not completely clear. Some studies have found that p53 exerts pro-autophagy and anti-autophagy effects under different conditions [26,27]. Under physiological conditions, the basal level of p53 (p53b) inhibits adenosine 5‘-monophosphate (AMP)-activated protein kinase (AMPK) to alleviate rapamycin complex 1 (mTORC1)-suppressed autophagy, which mTORC1 affects the formation of the ULK1/2 complex by interacting with FIP200. When stress occurs, activated p53 (p53a) translocates to the nucleus, where it promotes the transcription of autophagy genes (such as AMPK, PTEN, DRAM1, and Sestrins), and this p53-induced autophagy has no cardioprotective effect. Under high-stress conditions, when the intensity of pro-apoptotic stimuli exceeds the cell’s protective ability, the translocation of p53 to the mitochondria triggers mitochondrial outer membrane permeabilization, which is followed by the activation of the mitochondrial apoptotic pathway [26,27]. Interestingly, previous studies have shown that p53 does not have a direct effect on the expression levels of autophagy genes (ATG5, ATG12, and BECN-1). However, it indirectly contributes to autophagy and cell death in ventricular myocytes by directly altering mitochondrial membrane permeability [28] or causing mitochondrial interference via its downstream effector, the hypoxia-inducible death protein BNIP3 (Bcl-2/adenovirus E1B 19-kDa interacting protein 3) [21]. Moreover, Pan et al. demonstrated that miR-146a targeted TATA-binding protein (TBP) associated factor 9b (TAF9b) partially reverses DIC by indirectly destabilizing p53, inhibiting apoptosis, and regulating autophagy [29].

Mitophagy plays a role in the development of DIC by selectively removing structurally damaged and dysfunctional mitochondria [30]. Damaged mitochondria are cleared through the regulation of key mitochondrial fission and fusion proteins (Mfn1/2, OPA1 and DRP1) and mitophagy receptors (PINK1/Parkin, BNIP3/NIX, and FUNDC1) that directly or indirectly interact with the positive autophagosomal LC3, thereby contributing to mitochondrial encapsulation in autophagic vesicles to induce mitochondrial clearance [31,32]. The binding of cytoplasmic p53 to Parkin in DIC impairs the autophagic degradation of damaged mitochondria by inhibiting its mitochondrial transport [33]. Therefore, activating mitochondrial phagocytosis by inhibiting cytosolic p53 may be a potential therapeutic strategy for reducing DIC.

### 2.3. p53 Regulates Myocardial Atrophy

DOX exposure has been reported to reduce cardiac weight in mice [34]. Acute DOX-induced systolic dysfunction and reductions in heart weight and cardiomyocyte size have been found to be mediated by the p53-dependent inhibition of mammalian target of rapamycin (mTOR) signaling [35]. Similar DOX-induced adverse effects, including weakness, fatigue, dysfunction, and atrophy, have been observed in skeletal muscle tissue. The strong induction of p53/p21/REDD1 signaling is common to both effects. The downstream target genes in this axis induce cell cycle arrest and increase the levels of ROS, causing cardiac and skeletal muscle atrophy [36].

### 2.4. Upstream Targets Regulating p53 Protein Modification and Activity

A complex regulatory network upstream of p53 promotes its rapid activation during DOX stimulation. Multiple post-translational modifications (PTMs) have been shown to directly influence p53-induced target activation, and ultimately, p53-induced cell damage [37]. p53 phosphorylation is a key modification that exerts regulatory effects on apoptosis. In DIC, ROS and DNA damage activate the downstream checkpoint kinases Chk2 and Chk1 via ataxia telangiectasia mutated (ATM) and ataxia telangiectasia and Rad3-related (ATR) proteins, which together activate p53 phosphorylation. This promotes the split between p53 and MDM2 and allows transcription factors to bind to DNA [38]. The inhibition of Rac1, the major subunit of NADPH oxidase, enhances DIC via the ATM/p53/apoptotic pathway [39]. In addition, DOX activates mitogen-activated protein kinase (MAPK) signaling pathway components, including extracellular signal-regulated kinase 1/2 (ERK1/2), JNK, and p38, which then activate p53 phosphorylation and induce the mitochondrial apoptotic pathway [40]. The acetylation of p53, another important PTM, is essential for its activation, and it occurs through a reversible enzymatic process. Sirtuins (SIRT, class III histone deacetylases) such as SIRT1 [41,42], SIRT3 [43], and SIRT6 [44] have been shown to function as control points for several mechanisms in DIC [45]. Furthermore, Rac1 attenuates the DOX-mediated inhibition of histone deacetylase (HDAC), thereby ameliorating DIC [46]. Meanwhile, the ubiquitination of p53 is mutually exclusive with acetylation, and the ubiquitin E3 ligase ITCH degrades the ROS scavenger thioredoxin negative regulator, thereby inhibiting p38 MAPK/p53 pathway-mediated apoptosis in cardiomyocytes [47]. Apart from PTMs, Joiner et al. found that CaMKII induces apoptosis by promoting mitochondrial Ca^2+^ flow and p53, and the chaperone glucose-regulated protein 78 (GRP78) can restore DOX-induced cardiomyocyte apoptosis by inhibiting Ca^2+^-dependent CaMKII activation and p53 accumulation [48,49]. Interestingly, Huang et al. identified a novel stress-inducible protein, insulin-like growth factor receptor type IIα (IGF-IIRα), which activates the p53 apoptotic pathway [50].

## 3. Role of p53 in DOX Resistance

It has been established that p53 inhibits tumor formation and provides protection against DNA damage by inducing apoptosis, cell cycle arrest, and DNA repair. The overexpression of wtp53 promotes sensitivity to chemotherapeutic agents, while the expression of mutated p53 genes leads to DOX resistance. The involvement of p53 in the mechanism of chemoresistance has become a hot topic in recent reviews [51].

### 3.1. Mutant p53 (mutp53) Mediates DOX Resistance

p53 is the most commonly mutated gene in human cancers. The mutp53 gene has been identified in 42% of the 12 tumor types studied using cancer genome sequencing [52]. The majority of mutations occur in the core domain of p53, leading to a loss of DNA binding ability [50]. Mutp53 can also exhibit functional changes, such as loss-of-function (LOF), gain-of-function (GOF), or dominant negative effects [53]. Particularly, GOF mutations turn p53 into a proto-oncogene, which can further promote malignancy and chemotherapeutic resistance [53]. Furthermore, mutp53 is more stable than wtp53 because the binding of heat shock protein (HSP) 90 to mutp53 prevents its MDM2- and carboxy-terminus of Hsp70-interacting protein (CHIP)-mediated degradation following ubiquitination [54]. In addition, HSP70 and HSP40 have also been reported to stabilize mutp53 [55,56]. The various mechanisms of chemoresistance associated with p53 mutations have been extensively researched. These are summarized briefly in Figure 1b.

#### 3.1.1. Interaction between p53 and P-gp

The MDR transporter P-glycoprotein (P-gp, more commonly referred to as MDR1) can eliminate almost all drugs from within cells. It is the main cause of drug resistance in cancer cells. P-gp expression is most closely related to that of p53 [57]. As a transcriptional activator, wtp53 directly inhibits P-gp gene expression by binding to its head-to-tail p53 binding sequence and downregulating P-gp [58]. Moreover, ROR1, an upstream regulator of P-gp, is overexpressed in DOX-resistant breast cancers and regulates chemoresistance by modulating P-gp expression via ERK and p53 [59]. Adenovirus-mediated p53 overexpression can significantly reduce P-gp levels and increase DOX sensitivity [60]. Notably, mutp53 positively regulates P-gp expression in a variety of cancers, including osteosarcoma and breast, colorectal, and ovarian cancers [61,62]. For example, mutp53 (R248Q) can induce combined resistance against DOX and paclitaxel by upregulating P-gp [63]. Additionally, mutp53 accumulation in DOX-resistant MCF-7 cell lines increases P-gp expression [64]. These findings suggest that P-gp inhibitors are an effective tool for reducing or preventing MDR.

#### 3.1.2. Mutp53 Disrupts Cell Cycle Arrest and Apoptosis

In the presence of mutp53 expression or p53 deletions, tumor cells cannot be blocked in the G1 phase, abnormal cell proliferation increases, and apoptosis is inhibited, leading to drug resistance. DOX significantly activates caspase 3/7 in wtp53 cells, but not in migratory carcinoma cells expressing mutp53 [65]. In addition, unlike wtp53, mutp53 fails to activate BAX and downregulate Bcl-2 [51,66], and the overexpression of Bcl-2 can inhibit BAX and BAK activity, leading to DOX resistance [67]. Mutp53 (R273H) has been shown to induce cross-resistance to DOX and methotrexate by downregulating procaspase-3 [68]. Dominant-negative proteins or mutp53 also cause anti-apoptosis effects in tumor cells via the inhibition of p73 function [69]. Lin et al. revealed a novel mechanism in which mutp53 promotes intrinsic DOX resistance by downregulating miR-30c and subsequently upregulating the DNA damage repair proteins FANCY and REV1 [70].

#### 3.1.3. Mutp53 Increases CSC Differentiation

CSCs are a rare subpopulation of tumor cells with the ability to self-renew and multiply differentiate, and are thought to be the cause of drug-resistant relapse after chemotherapy [71]. Although DOX kills most rapidly proliferating tumor cells, numerous preclinical and clinical studies have shown that DOX resistance is often associated with CSC enrichment and that DOX is largely ineffective at eliminating CSCs [72]. Wtp53, a potent stem cell inhibitor, prevents suboptimal cells that carry DNA damage from becoming iPSCs by inducing their apoptotic elimination [73]. Functional p53 also inhibits the epithelial–mesenchymal transition (EMT), and escape from chemotherapeutic toxicity through EMT acquisition is an essential feature of CSCs [74,75]. Recent studies have revealed a close association between p53 and EMT, in which the mutational status of p53 decides the outcome of the EMT process [76]. In contrast to wtp53, which negatively regulates EMT, mutp53 can drive part of the EMT process via the EMT transcription factors Twist and Slug [77]. Indeed, in addition to regulating the EMT program, p53 can also modulate the extracellular matrix (ECM). Accumulating evidence has demonstrated that ECM remodeling promotes the development and maintenance of stemness and chemoresistance [78,79,80,81]. ECM is composed of collagen, non-collagenous glycoproteins (mainly fibronectin and laminin), and proteoglycans. ECM components broadly regulate cellular processes and functions, including cell proliferation and survival, migration, differentiation, autophagy, angiogenesis, and immune regulation [82]. There is growing evidence that mutp53 promotes tumor neovascularization and regulates the secretion of secreted proteins and chemokines [83]. Mechanistically, p53 LOF is associated with the upregulation of ECM components (e.g., fibronectin, extracellular matrix metalloproteinase inducer, and matrix metalloproteinases), and these changes are accompanied by invasion-metastasis and the inhibition of apoptosis in tumor cells [79,84]. Notably, mutp53 expression is often associated with a high incidence of CSCs in tumors, and mutp53 GOF contributes to their development [85]. The expression of iPSCs generated by p53 mutant precursors is considered a marker gene for CSCs, and confers DOX resistance to these cells.

##### 3.1.4. m^6^A Methylation of mutp53

m^6^A RNA methylation is the most common internal modification in mammalian mRNAs, and lncRNAs that regulate oncogenic signaling pathways contribute to carcinogenesis [86]. The Gb3-cSrc complex within sphingolipid-enriched microdomains in cancer cell membranes can upregulate m6A modifications in the p53 pre-mRNA point mutation codon 273 by increasing the expression of methyltransferase-like 3, producing DOX resistance with increasing ceramide glycosylation [87]. The inhibition of ceramide glycosylation suppresses m^6^A methylation in p53 pre-mRNA, activates the function of the wtp53 gene, and restores the sensitivity of drug-resistant cancer cells to DOX [88].

### 3.2. Degradation and Inactivation of wtp53

The inactivation or inhibition of certain p53 functions is believed to be a prerequisite for chemoresistance in most human cancers. Lin et al. reported that RNA-binding protein 28 inhibits p53 transcription by interacting with the DNA-binding structural domain of the p53 gene via Chk1/Chk2 translocation from the nucleolus to the nucleoplasm, further explaining the stronger DOX resistance seen in wtp53 cancer cells [89]. Below, we summarize and describe the mechanisms associated with wtp53 in DOX-induced tumor drug resistance.

#### 3.2.1. Mechanism of Regulating wtp53 Degradation

p53 ubiquitination and its subsequent degradation can affect the response to DOX therapy and promote tumor formation, progression, and chemoresistance. In 10–20% of human cancers, this occurs due to the inactivation of p53 ubiquitination-mediated degradation via the amplification of MDM2 or MDM4 [90]. MDM2-mediated p53 degradation is a key factor in DOX-acquired resistance in HepG2 cells. By developing small-molecule inhibitors or degraders that block the interaction between p53 and MDM2, the oncogenic activity of p53 can be restored in patients carrying wtp53 [91]. Other deubiquitinases have also been implicated in the regulation of p53 during DOX resistance. For example, OTU dismutase 5 (OTUD5) and C-Jun activation domain-binding protein 1 (JAB1) enhance DOX and cisplatin resistance via p53 ubiquitination [92,93]. Ribosomal L1 structural domain protein 1 (RSL1D1) has been identified as a novel negative regulator of p53 in human colorectal cancer cells. Through direct interaction, it inhibits the E3 ligase activity of HDM2, enhancing the ubiquitination-mediated degradation of p53 and decreasing p53 protein levels [94]. The oncogene Gankyrin (Gank), a key molecule in hepatoblastoma, induces tumor growth by triggering the degradation of p53 [95]. Mechanistically, Gank interacts with MDM2 to amplify MDM2-mediated p53 poly-ubiquitylation, leading to p53 degradation. Additionally, the hyper-phosphorylated retinoblastoma protein (pRb) stabilizes p53 by inhibiting the interaction of the Gank-MDM2-p53 complex via binding to the central domain of MDM2. Gank and MDM2 overexpression leads to pRb inactivation, which, in turn, enhances the formation of the Gank-MDM2-p53 complex and subsequent p53 degradation [96].

#### 3.2.2. Wtp53 and Non-Coding RNAs (ncRNAs)

NcRNAs, including microRNAs, lncRNAs, piRNAs, and circular RNAs, can regulate the therapeutic sensitivity of cancer cells [97]. Hence, they can play a role in the acquisition of drug resistance. In recent years, accumulating evidence has shown that various miRNAs, including miR-125b, miR-504, miR-25, miR-30d, miR-1285, miR-27a, and miR-380-5p, can downregulate p53 protein levels [98,99]. The overexpression of the lncRNA LINP1 is positively associated with proliferation, drug resistance, and metastasis in breast cancer cells. LINP1 knockdown can promote BC cell metastasis and improve resistance to DOX by blocking the effects of p53 [100]. Compared with microRNAs and lncRNAs, piRNAs have been studied to a lower extent in this field. By screening for piRNA-36712, a novel tumor suppressor, using cancer genome atlas data analysis, researchers found that piRNA-36712 expression is significantly lower in breast cancer cells than in normal breast tissues. Moreover, this piRNA suppresses p53 through high selenoprotein W pseudogene expression, thus promoting the proliferation, invasion, and migration of cancer cells. Moreover, piRNA-36712 also produces synergistic anticancer effects with paclitaxel and DOX [101].

#### 3.2.3. Tumor Microenvironment Regulates wtp53

The tumor microenvironment refers to the internal environment wherein tumor cells arise and survive. It contains cancer-associated fibroblasts (CAFs), ECM, microvasculature, and various cytokines and chemokines that can modulate chemosensitivity [102,103]. The ECM and CAFs form a protective barrier, preventing drug diffusion and activating resistance pathways through anti-apoptotic effects and microenvironmental remodeling (e.g., hypoxia and metabolic stress) [81]. When cancer cells are co-cultured with CAFs or in CAF-conditioned medium, DNA damage and the p53-mediated response to chemotherapy drugs are reduced, and drug resistance is enhanced via the inhibition of drug accumulation and the antagonization of drug-induced oxidative stress [104]. Moreover, the interleukin-6 (IL-6) produced by CAFs can reduce the response of p53 to DOX [105]. In addition to increasing the levels of MRPs and anti-apoptotic proteins, chronic exposure to DOX induces an MDR phenotype in drug-resistant MCF-7 cells through the upregulation of the ECM glycoprotein pp-GalNAc-T6 (which mainly constitutes carcinoembryonic fibronectin) [80]. The integrin-dependent downregulation of p53 levels in melanoma, sarcoma, and fibroblasts results in increased cell survival and resistance to apoptosis [106]. Furthermore, in breast cancer cells, ECM stiffness affects the p53 activation induced by DOX. A soft ECM downregulates RHO kinase 2 (ROCK2), which drives chemoresistance by inhibiting p53 activation [107]. EMT plays an important role in cancer cell plasticity, leading to tumor heterogeneity. Studies have shown that activation of the EMT program plays a key role in drug resistance by converting non-CSCs to CSCs. Leucine-rich repeat-containing G protein-coupled receptor 5 (Lgr5), an EMT inducer, promotes DOX resistance by regulating the programmed cell death 5 (PDCD5)/p53 pathway to inhibit apoptosis [108]. Tight junctions (TJs) maintain cell polarity, permeability, and adhesion and participate in the regulation of cell proliferation and differentiation. The Claudin (CLDN) family is an integral part of TJs. CLDN6 is mainly expressed in tumor tissues. CLDN6 interacts with p53 and promotes its translocation from the nucleus to the cytoplasm, where p53 positively regulates glutathione S-transferase-p1 (GSTP1) to promote DOX resistance in MCF-7 cells [109]. Hypoxia, a common feature of all solid tumors, can downregulate p53 and promote vascularization, EMT, mobility, and metastasis in cancer cells. Further, it can alter metabolism and promote resistance to anticancer therapies [110].

## 4. Nutrients Can Attenuate DOX Toxicity and Drug Resistance by Targeting p53

**Figure 2:**
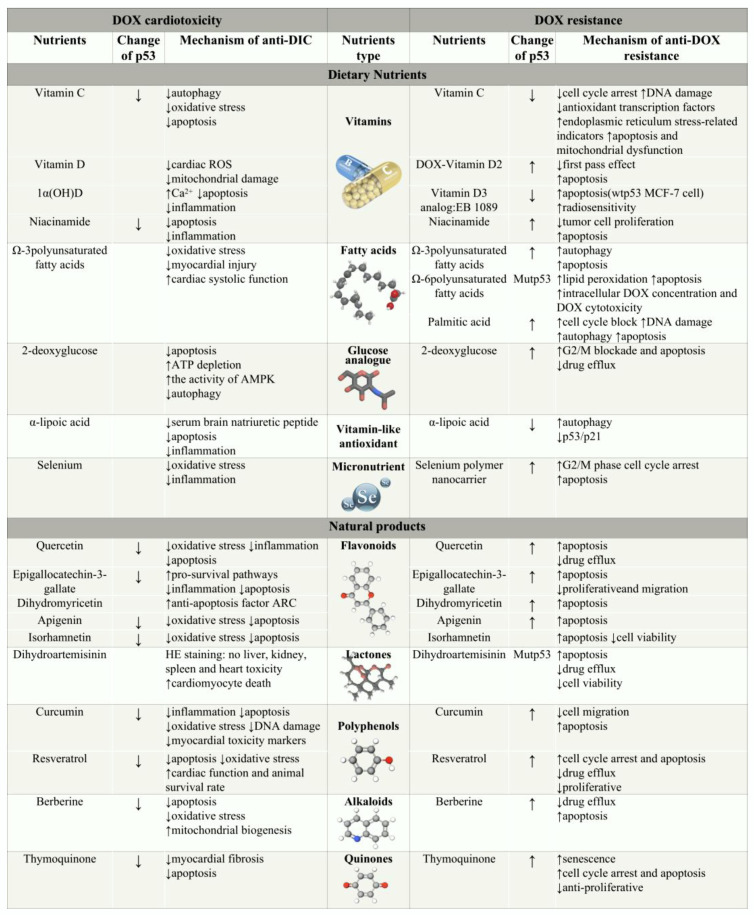
Mechanism by which nutrients overcome DOX cardiotoxicity and resistance by targeting p53. ↑Arrows indicate upregulation or facilitation; ↓Arrows indicate downregulation or suppression.

There are two major obstacles that limit the clinical application of DOX: tumor drug resistance and cardiotoxicity (Figure 1). Hence, researchers have attempted to use the dual model of cardiac cytotoxicity/chemotherapeutic efficacy to screen effective chemotherapeutic adjuvants that can be administered in combination with DOX. Figure 2 summarizes recent findings on the use of various dietary nutrients and natural products for preventing DOX toxicity and resistance via p53 modulation. Their differential effects on p53 in different settings indicate different cellular outcomes, which may explain the possible mechanisms underlying these effects.

### 4.1. Dietary Nutrients

In recent years, antitumor dietary therapy has become an important approach in cancer treatment. Some dietary nutrients and their derivatives have been experimentally shown to increase DOX antitumor activity and are considered safe and convenient as supplements to established therapies. Dietary changes do not alter nutrient levels in tumors and healthy tissues in a balanced manner. Further, similar to new drug combinations, the combination of dietary nutrients and DOX must be adapted based on the specific tumor and its metabolic activity, and clinical trials are required before an optimal anticancer regimen may be identified [111]. Here, we summarize the specific mechanisms by which vitamins, fatty acids, glucose analogs, and the micronutrient selenium regulate DIC and DOX resistance via p53.

#### 4.1.1. Vitamins

Vitamin C (L-ascorbic acid) is a well-known antioxidant, and several studies have investigated the beneficial effects of vitamin C in combination with DOX [112]. One of these studies noted that that low concentrations of L-ascorbic acid (below 7 mM) can downregulate some apoptotic, cell survival-, and cell cycle-related proteins, without affecting the RNA levels of p53, ultimately increasing the sensitivity of HeLa cells to cisplatin and DOX via the induction of a DNA damage response [113]. Significant attenuation of DIC via the inhibition of apoptotic proteins and antioxidants was also observed in isolated rat cardiomyocytes pretreated with vitamin C [114].

Vitamin D is a fat-soluble vitamin. Vitamin D2 coupled with DOX (VitD-DOX) has emerged as a novel DOX administration modality. The treatment of human osteosarcoma cells with 10 µM VitD-DOX for 24 h was found to significantly activate the expression of p53 [115]. Likewise, vitamin D3 analogue EB 1089 could interfere with MAPK activity and promote apoptosis in wtp53 MCF-7 cells when administered in combination with DOX, but it was ineffective in mutp53 MCF-7 cells [116]. Furthermore, in female mice with triple-negative mammary tumors, vitamin D supplementation (10,000 IU/kg) was found to reduce DIC in vivo by reducing ROS and mitochondrial damage without decreasing the anticancer effects of DOX [117].

Nicotinamide (NAM) belongs to the vitamin B group. Studies have shown that exogenous NAM selectively inhibits the proliferation of DOX-resistant K562 cells and promotes apoptosis by activating p53 [118,119]. A recent comparative study revealed that the administration of NAM (600 mg/kg) and the active vitamin D analogue 1α(OH)D3 (0.5 μg/kg) for 4 weeks effectively reverses DIC in rats. Mechanistically, the combination of NAM and vitamin D prevented the myocardial mitochondria-mediated apoptotic cascade by maintaining Ca^2+^ endostasis. In addition, the NAM-induced blockade of inflammatory signaling provides a better cardioprotective effect than 1α(OH)D3 [120].

α-lipoic acid (LA) is called a “super antioxidant” and is a vitamin-like water- and fat-soluble metabolic antioxidant. Mutp53 degradation may attenuate its pro-tumor and metastatic activity. A recent clinical study showed that LA treatment (600 mg daily) can significantly decrease oxidative stress and inflammatory markers [121]. In this context, LA may enable supportive treatment for cancer and may represent a promising adjuvant therapy for mitigating DIC.

#### 4.1.2. Glucose Analogs

Cancer cells use glycolysis as their major mode of energy metabolism, and this metabolic phenotype is an important factor in the progression of MDR to DOX [122]. The glucose analogue 2-deoxy-D-glucose (2-DG) antagonizes DIC by mimicking the effects of caloric restriction at the cellular level. It achieves this by blocking ATP depletion, inhibiting myocardial apoptosis and AMPK activation, and inhibiting DOX-induced deleterious autophagy, without affecting caloric intake [123]. 2-DG significantly inhibits drug resistance in MCF-7 cells via the upregulation of p53 and downregulation of P-gp [122]. In addition, the combination of metformin and 2-DG can reverse drug resistance in MCF-7 cells by restoring p53 function and increasing DOX accumulation through the inhibition of murine MDM2 and MDM4 [124]. The rational design of energy inhibitors or caloric restriction mimetics for DOX and tumor cells’ energy metabolism characteristics warrants further investigation.

#### 4.1.3. Fatty Acids

The dysregulation of lipid metabolism is one of the most prominent metabolic alterations in cancer, and many experts believe that saturated fatty acids are an important cause of human cancers. Treatment with the saturated fatty acids palmitic acid, stearic acid, and myristic acid reduces the accumulation of p53 and promotes tumorigenesis [125]. In contrast, physiological concentrations of exogenous palmitic acid (10–200 μM) can act as adjuvants in endometrial cancer treatment, potentially by significantly inhibiting the cell cycle blockade proteins FAS, p53, and Cyclin D1 and inducing DNA damage, autophagy, and apoptosis [126]. Different polyunsaturated fatty acids (PUFAs) have different effects on the treatment of malignancies. Epidemiological evidence suggests that Ω-6 PUFAs promote cancer, while Ω-3 PUFAs prevent it [127]. Jing et al. found that Ω-3 PUFA treatment results in autophagy via p53-mediated AMPK/mTOR signaling and sensitizes tumor cells to apoptosis [128]. In addition, a population study showed that long-term supplementation with Ω-3 PUFA attenuates DIC in pediatric patients with acute lymphoblastic leukemia [129]. In contrast, Bose et al. proposed that Ω-6 PUFAs have a diametrically opposite effect in cancer prevention and treatment. In their study, the intake of Ω-6 PUFAs increased the formation of the metabolite 4-hydroxynonenal, increased lipid peroxidation, and improved DOX chemosensitivity in mutp53 breast cancer cells [127].

#### 4.1.4. Micronutrients

The micronutrient selenium inhibits thioredoxin reductase activity, thus interfering with oxidative stress. As a result, it boosts the efficacy of anticancer drugs and is considered as an adjuvant to chemotherapy [130]. However, large doses (cumulative 1.3 g) of selenium can produce serious toxicity [131]. Therefore, selenium polymer nanocarriers were recently developed to induce cell cycle arrest and p53-mediated caspase-independent apoptosis in order to arrest cancer cell proliferation, which could reduce the cytotoxicity of many chemotherapeutic drugs, including DOX [130]. Notably, mice fed selenium-supplemented diets showed significantly lower DOX-induced oxidative damage and inflammation in cardiac tissues. In other words, controlled selenium supplementation and the development of nanoformulations could help in the application of selenium in adjuvant strategies for chemotherapy [132].

### 4.2. Natural Products

The wide-ranging pharmacological properties of natural products have encouraged scientists to seek effective natural substances, or their more effective derivatives or prodrugs, as adjuvants to DOX treatment [133]. Natural products are typically considered to be relatively inexpensive, less toxic, and easy to use. Flavonoids, sesquiterpenoids, alkaloids, diterpenoids, and saponins have all demonstrated excellent potential in combating the toxicity and adverse effects of DOX.

Flavonoids are a class of compounds with a 2-phenyl chromogenic ketone structure and are widely found in nature [134]. Quercetin (QUE), the most abundant dietary flavonol, shows excellent efficacy in combination with DOX due to its potent biological activity. On the one hand, QUE significantly increases the sensitivity of pancreatic cancer and hepatocellular carcinoma cells to gemcitabine and DOX by decreasing hypoxia-inducible factor-1α (HIF-1α) expression and inhibiting P-gp [135]. On the other hand, the anti-inflammatory and antioxidant activity of QUE provides protective effects to normal cells. QUE selectively enhances the toxic effects of DOX against hepatocellular carcinoma cells without damaging normal hepatocytes [136]. In a mouse oral QUE model and H9c2 cells, QUE was found to reduce oxidative stress and myocardial apoptosis by downregulating p53 and NADPH oxidase 1 [137]. The combination of QUE and DOX also provided p53-related hepatoprotective effects to the liver and immune system in a rat model [138,139].

Epigallocatechin-3-gallate (EGCG) is the most abundant and biologically active flavanole in tea and is widely used as a health-promoting food ingredient. EGCG in combination with DOX can inhibit bladder cancer cell proliferation by increasing p53 expression [140]. In a DIC model, EGCG (40 mg/kg) pretreatment for 4 weeks exerted anti-inflammatory and apoptotic effects on heart cells by inhibiting the expression of Nuclear factor-κB (NF-κB), p53, caspase 3, and caspase 12 [141].

Dihydromyricetin (DHM) is a special flavonoid compound obtained from vine tea (*ampelopsis grossedentata*). DHM (25 μM) can increase the sensitivity of drug-resistant human ovarian cancer cells to paclitaxel and DOX via the p53-mediated downregulation of survivin [142]. DHM (50 μM) was also found to attenuate cardiomyocyte proliferation arrest and apoptosis by regulating MDM2 and promoting the accumulation of ARC, an important anti-apoptotic factor, in H9c2 cells [143].

Apigenin is a naturally occurring flavone and is also known as “phytoestrogen”. The synergistic anticancer activity of apigenin and DOX in K562 cells was linked to the increased expression of p53 and downregulation of BAX and Bcl-2 [144]. Apigenin (100 mg/kg) not only reduced DIC in Wistar rats by modulating the p38/JNK/p53 signaling pathway, but also significantly upregulated antioxidant responses [145].

Isorhamnetin is a promising and novel antitumor flavonoid extracted from the sea-buckthorn (*Hippophae rhamnoides* L.). When co-administered with DOX, it was found to decrease viability and increase apoptosis in MCF-7, HepG2, and Hep2 cells, and these effects were more prominent than those in the DOX alone group [146]. Isorhamnetin has also been demonstrated to provide antioxidant effects against DOX-induced oxidative stress and to inhibit the mitochondrial apoptosis (decreasing p53) pathway [146].

Dihydroartemisinin contains peroxisomal sesquiterpene lactones with potent antimalarial and anticancer activities. The combination of dihydroartemisinin and DOX at an optimal concentration of 10 µg/mL increases chemosensitivity to DOX [147]. In hepatocellular carcinoma cells expressing mutp53 (R248Q), dihydroartemisinin can reduce P-gp expression and restore DOX sensitivity by inhibiting the p53/ERK1/2/NF-κB signaling pathway [63]. In an animal study, no toxic side effects were observed in the livers, kidneys, spleens, or hearts of tumor-bearing mice treated with dihydroartemisinin (15 mg/kg) [147]. In contrast, dihydroartemisinin was reported to be a pharmacological translational control tumor protein inhibitor (30 mg/kg), inducing heart failure and cardiomyocyte death in mice following continuous intraperitoneal injection for 4 weeks. Hence, its dose-dependent toxicity still needs further investigation [148].

Curcumin (CUR) and resveratrol (RES) are natural polyphenol compounds with multiple activities, including anti-inflammatory, antioxidant, anti-proliferative, and anti-angiogenic effects. CUR is classified by the National Cancer Institute as a third-generation oncotherapeutic agent [149], with a unique role as a p300 histone acetyltransferase inhibitor in DOX-resistant cancer cells. Thus, it can enhance the transcriptional activity of p53 and endogenous caspase cascade activation by relieving the inhibition of the NF-κB pathway, and thus, triggering p53-p300 crosstalk [150]. Furthermore, CUR (below 50 μM) treatment induces p53/p21-dependent apoptosis and significantly inhibits SH-SY5Y cell growth and migration [151]. An in vitro study showed that CUR treatment (200 mg/kg/day, gavage) can significantly downregulate the expression of genes such as Rac1 and p53 in myocardial tissue, highlighting a potential application of CUR in the prevention of DIC [152]. Meanwhile, RES, an anthraquinone terpenoid that is highly abundant in grapes, was first reported to have an anticancer effect in 1997 [153]. In recent years, the mechanism underlying the inhibitory effects of RES and DOX on the production, proliferation, and metastasis of various tumor cells has been discovered. For example, RES (10 μg/mL) triggered growth inhibition in lymphocytic leukemia cell lines through the hyperactivation of phosphorylation at the phosphatase and tensin homolog (PTEN) and p53 ser15 [154]. RES (50–100 µM) treatment increased the expression of both BAX and Bcl-2 and inhibited P-gp expression in HepG2 and MCF-7 cell lines, while upregulating p53 only marginally [155]. Further, the effects of RES treatment (10–100 μM) on DOX B16 melanoma cell subline growth were shown to be induced by increased p53 expression levels [156]. In normal cells, RES acted as a SIRT1 agonist and improved cardiac function and survival by restoring SIRT1 activity, thereby reducing p53 acetylation. RES attenuated USP7, a p53 deubiquitinating protein, and inhibited the pro-apoptotic markers p53, BAX, and caspase 3 by activating SIRT1 [157].

Berberine (BER), mainly obtained from the Chinese herbal medicine Huanglian, is a quaternary alkaloid with yellow needle-like crystals. Daily administration of BER (5 mg/kg) via oral gavage can enhance sensitivity to DOX by inhibiting P-gp [158]. Moreover, high-dose BER (200 mg/kg) directly induces apoptosis in cancer cells by activating the AMPK/p53 pathway [158]. In addition, BER induces autophagy and apoptosis in p53-negative leukemia cells in a non-p53-dependent manner by down-regulating the expression of MDM2 at the transcriptional and post-transcriptional levels [159]. Notably, BER pretreatment promotes p53 deacetylation via SIRT1/3 levels, thereby preventing cardiomyocyte apoptosis, providing a promising approach against DIC [160].

Thymoquinone (TQ), a quinone isolated from black seed (*Nigella sativa*), has been demonstrated to have anticancer properties. TQ stimulates ROS to a greater extent in mutp53 cells than in wtp53 cells, and this could be because the activation of p53 induces cell cycle arrest to repair TQ-induced damage [161]. TQ upregulates the transcriptional levels of PTEN, a tumor suppressor that exerts anti-proliferative effects, leading to the phosphorylation of p53/p21. Thus, it induces a G2/M phase block and apoptosis in MCF-7 cells [162]. Moreover, El-Far et al. showed that combined treatment with TQ (50 µM), CUR (15 µM), and caffeine (10 mM) can result in significant upregulation of the senescence marker β-galactosidase, as well as p53/p21, increasing DOX efficacy [163]. Correspondingly, the administration of 50 mg/kg TQ for 8 weeks in rats inhibits DOX-induced apoptosis and fibrosis, suggesting that TQ may be a potential therapeutic agent for reducing DIC [164].

Even though some molecules have shown promise, due to the potential of unknown toxicity, low bioavailability, and DOX interactions, clinical studies are required to validate the results seen in preclinical models. Subsequent studies should concentrate on the synergistic effects of natural products in combination with DOX.

## 5. Dual-Drug Strategy to Overcome both DOX Resistance and Cardiotoxic Side Effects by Targeting p53

**Figure 3:**
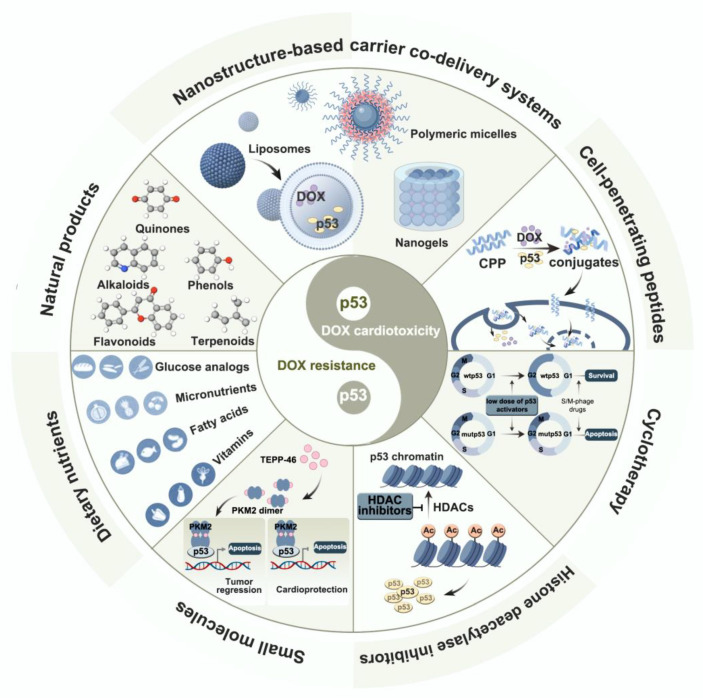
p53-targeted approaches to combating DOX cardiotoxicity and medication resistance. It has been demonstrated that a variety of nanostructure-based carrier co-delivery systems, cell-penetrating peptides (CPPs), circulating therapies, histone deacetylase (HDAC) inhibitors, endogenous small molecules, dietary nutrients, and natural products can increase the sensitivity of tumor cells to DOX by modulating p53 activity, and these tactics also have cardioprotective effects.

Given that p53 is a key nexus for various tumor suppression pathways, extensive research has been devoted to determining how p53 function can be restored in human cancers in order to reduce cellular resistance to DOX therapy. Many anti-DIC therapeutic strategies have also been investigated to gain cardioprotective effects by inhibiting p53 activity. To date, in addition to natural products and nutrients, several DOX dual-drug strategies have been developed to improve chemosensitivity while reducing DOX dose and improving its efficacy, as summarized and discussed in Figure 3.

In recent years, nanotechnology-based carrier co-delivery systems have attracted great interest for the delivery of chemotherapeutic drugs [165]. DOX nano-formulations (e.g., liposomes [166], polymeric micelles [167], and DOX nanogels [168]) not only promote drug accumulation in cancer cells through enhanced permeation and retention, but also minimize non-specific DOX toxicity due to their unique molecular structure. This novel and simple model of direct intervention during DOX release control and delivery can directly increase p53 levels and reverse MDR, and has significant translational potential. In addition, DOX can be directly co-delivered with p53, other chemotherapeutic agents (e.g., methotrexate and rapamycin) [169], or MDR reversal agents (e.g., efflux pump inhibitors or redox cell state modulators) via nanocarriers to directly or indirectly synergistically induce cancer cell apoptosis by increasing p53 [167,170,171]. However, most drugs are still in the research stage. This is because there are various limitations to the clinical application of nanoparticle-mediated therapy, including high production costs, batch inconsistency in nanoparticles, and unformulated test protocols and models.

Similar to nanosystems, cell-penetrating peptides (CPPs) can bind to conventional chemotherapeutic drugs such as DOX, enabling preferential DOX uptake and growth inhibition. They can also be directly coupled to p53, leading to p53 overexpression in tumor cells [172]. The use of CPPs is limited by issues such as dose toxicity, poor water solubility, and immunogenic resistance, and more studies and clinical trials are required to address these drawbacks and test their effects on different tumor types [173].

Cyclotherapy is an emerging therapeutic strategy. In p53 gene-based cyclotherapies, the apoptosis of mutp53 cancer cells can be specifically achieved using low doses of p53 gene activators, such as nutlin-3 and actinomycin D, without affecting the cell cycle in normal cells carrying wtp53 [174].

In recent years, epigenetic mechanisms have become a hot topic in the development of antitumor drugs, and HDAC inhibitors (HDACi) are the first class of epigenetic drugs approved for tumor treatment [175]. In cancer cells, HDAC overexpression leads to enhanced histone deacetylation in the p53 gene, making the nucleosomes compact and decreasing p53 gene expression. HDAC inhibition may promote cell death via p53 deacetylation. Clinical trials have reported the synergistic effects of HDACi and DOX combination therapy [176], and studies using animal models have found that butyroyloxymethyl not only increases the anticancer activity of DOX, but also exerts cardioprotective effects [177]. However, Sonnemann et al. demonstrated that the HDACi vorinostat and entinostat exhibit p53-independent cytotoxic activity [178]. Therefore, there is a need to delineate the mechanisms of HDACi and optimize anticancer regimens to make them faster and more effective for clinical use.

In addition to exogenous agents, endogenous small molecules play multiple interesting roles in antitumor effects and protection against DOX toxicity. The redox-metabolizing enzyme pyruvate kinase 2 (PKM2) has been a hot topic of research in recent years [179]. Saleme et al. used PKM2 to treat mice with lung tumors and found that PKM2 combined with DOX could activate p53 expression in tumor tissues and induce tumor cell death. By taking advantage of the differences in oxygen tension between the myocardium and tumors, PKM2 could inhibit the activity of the apoptotic protein p53 in myocardial tissues, thus preventing the apoptosis of myocardial cells while enhancing tumor regression in a lung cancer model [180]. Thus, PKM2 could be a new target for enhancing the safety and efficacy of DOX chemotherapy, but further experimental and clinical studies are needed to determine its safety and suitability.

## 6. Conclusions and Future Perspectives

As a tumor inhibitor, p53 greatly reduces the efficacy of chemotherapy following its extensive mutation and degradation inactivation. This is one of the main reasons for DOX resistance. Therefore, p53 reactivation is a promising strategy to overcome DOX resistance. It is widely accepted that the inhibition of p53 attenuates DOX-induced damage in non-cancer cells [181,182], but we cannot ignore that inhibiting p53 itself can promote tumor growth and reduce the antitumor activity of DOX. Owing to the potent antitumor efficacy of p53, which inevitably leads to the death of non-cancer cells, and polymorphisms of the p53 gene in the tumor environment, antitumor strategies targeting p53 are an attractive option for improving the clinical utility of DOX. However, this appears to be challenging due to the complexity of p53 gene polymorphisms and regulatory networks [85]. In general, the dynamic and interdisciplinary exchange of knowledge is required among biologists, chemists, materials scientists, and oncologists. Closer collaboration between academia, the pharmaceutical industry, clinical institutions, and regulatory agencies would also support this goal. Combining diverse and complementary expertise will be crucial for successfully addressing the toxicity and resistance challenges in the application of DOX and for better leveraging its antitumor activity.

## Data Availability

Not applicable.
